# Development and Evaluation of a Blood Culture PCR Assay for Rapid Detection of *Salmonella* Paratyphi A in Clinical Samples

**DOI:** 10.1371/journal.pone.0150576

**Published:** 2016-03-01

**Authors:** Liqing Zhou, Claire Jones, Malick M. Gibani, Hazel Dobinson, Helena Thomaides-Brears, Sonu Shrestha, Christoph J. Blohmke, Thomas C. Darton, Andrew J. Pollard

**Affiliations:** Oxford Vaccine Group, Department of Paediatrics, University of Oxford and the NIHR Oxford Biomedical Research Centre, Oxford, United Kingdom; Universidad Nacional de la Plata, ARGENTINA

## Abstract

**Background:**

Enteric fever remains an important cause of morbidity in many low-income countries and *Salmonella* Paratyphi A has emerged as the aetiological agent in an increasing proportion of cases. Lack of adequate diagnostics hinders early diagnosis and prompt treatment of both typhoid and paratyphoid but development of assays to identify paratyphoid has been particularly neglected. Here we describe the development of a rapid and sensitive blood culture PCR method for detection of *Salmonella* Paratyphi A from blood, potentially allowing for appropriate diagnosis and antimicrobial treatment to be initiated on the same day.

**Methods:**

Venous blood samples from volunteers experimentally challenged orally with *Salmonella* Paratyphi A, who subsequently developed paratyphoid, were taken on the day of diagnosis; 10 ml for quantitative blood culture and automated blood culture, and 5 ml for blood culture PCR. In the latter assay, bacteria were grown in tryptone soy broth containing 2.4% ox bile and micrococcal nuclease for 5 hours (37°C) before bacterial DNA was isolated for PCR detection targeting the *fliC-a* gene of *Salmonella* Paratyphi A.

**Results:**

An optimized broth containing 2.4% ox bile and micrococcal nuclease, as well as a PCR test was developed for a blood culture PCR assay of *Salmonella* Paratyphi A. The volunteers diagnosed with paratyphoid had a median bacterial burden of 1 (range 0.1–6.9) CFU/ml blood. All the blood culture PCR positive cases where a positive bacterial growth was shown by quantitative blood culture had a bacterial burden of ≥ 0.3 CFU/ ml blood. The blood culture PCR assay identified an equal number of positive cases as automated blood culture at higher bacterial loads (≥0.3 CFU/ml blood), but utilized only half the volume of specimens.

**Conclusions:**

The blood culture PCR method for detection of *Salmonella* Paratyphi A can be completed within 9 hours and offers the potential for same-day diagnosis of enteric fever. Using 5 ml blood, it exhibited a lower limit of detection equal to 0.3 CFU/ml blood, and it performed at least as well as automated blood culture at higher bacterial loads (≥0.3 CFU/ml blood) of clinical specimens despite using half the volume of blood. The findings warrant its further study in endemic populations with a potential use as a novel diagnostic which fills the present gap of paratyphoid diagnostics.

## Introduction

Enteric fever is a systemic illness caused by infection with *Salmonella enterica* serovars Typhi and Paratyphi. It remains a leading cause of morbidity worldwide [1,2]. Historically, *Salmonella* Typhi (*S*. Typhi) is thought to have accounted for the majority of the global burden of enteric fever. Recent evidence suggests that *Salmonella* Paratyphi A (*S*. Paratyphi A) is the aetiological agent in an increasing proportion of cases, particularly in South East Asia and the Indian sub-continent [1,3]. It was estimated that 26.9 million and 4.9 million cases of *S*. Typhi and *S*. Paratyphi A, respectively, occurred globally in 2010 [4]. Prevention of enteric fever requires effective control of both *S*. Typhi and *S*. Paratyphi.

The control of enteric fever is hampered by the lack of accurate estimates of disease burden, which stems, in part, from the absence of highly sensitive and specific diagnositic tests. Current diagnostics include the Widal test, developed in 1896, and several other serologically based assays, such as Typhidot and Tubex [5,6], which are based on detection of antibodies in blood. Such tests suffer from low sensitivity and specificity, resulting from cross-reactivity of antibodies induced by conserved antigens of the Enterobacteriaceae, as well an inability to accurately distinguish current and previous infection. Automated blood culture is currently the mainstay of diagnosis of enteric fever and the ‘gold-standard’ against which any novel diagnostic is compared. The sensitivity of blood culture has been variably reported at 40–80%, with higher sensitivity in the first week of illness, when the bacterial concentration in blood is an order of magnitude higher than in subsequent weeks [7]. Other important limitations of automated blood culture include the requirement for dedicated facilities and trained staff, which limits its use in resource limited settings. In addition, automated blood culture is time-consuming and may take several days for isolation and identification of causative organisms. Whilst continuing efforts are concentrated on developing novel tests for *S*. Typhi, the development of diagnostics to specifically identify cases of *S*. Paratyphi A is lacking. This is of clinical importance as *S*. Paratyphi A appears to be responsible for an increasing proportion of enteric fever cases. The availability of diagnostics able to discriminate the enteric fever-causing *Salmonella* species is also vital, prior to the roll-out of mono or polyvalent vaccines in order to assess the differential contribution of vaccine efficacy in disease prevention, and to recognise possible ‘serovar replacement’.

Molecular methods, especially polymerase chain reaction (PCR) based assays, have attracted much attention in last decade for diagnosis of enteric fever [8]. The low bacterial burden in blood of enteric fever patients (estimated as a median of 0.3 CFU/ml blood [9]) hinders the technical progress in this field. Several studies on the use of PCR or nested PCR reported good sensitivity and specificity when compared to blood culture proven cases and healthy controls [10–17]. However, the practical utility of PCR tests in the clinical setting needs to be further evaluated. Recently, Tennant *et al*. [18] developed a clyA-based real time PCR method to detect *S*. Typhi and *S*. Paratyphi A simultaneously in blood, but in field test it was only 40% as sensitive as blood culture. When template DNA is prepared from specimens containing a low bacterial load, such as occurs in the blood of enteric fever patients, it is dominated by the background host blood DNA, which swamps the bacterial target signal leading to either false-positive PCR results (due to the non-specific binding of primer) or false-negative results due to reduced sensitivity [19–21]. In order to overcome this limitation, we and others have previously demonstrated the utility of a pre-culture incubation step prior to PCR amplification [22,23]. This approach has proved valuable for early diagnosis of typhoid fever [8].

Herein, we describe the development and optimisation of a novel blood culture PCR assay for detection of *S*. Paratyphi A, and the evaluation of its performance using blood samples derived from volunteers experimentally challenged with *S*. Paratyphi A [24]. By applying this novel blood culture PCR assay, we are able to accurately diagnose paratyphoid infection in samples with a bacterial load ≥0.3 CFU/ml. We demonstrate that blood culture PCR can be completed within 9 hours of sample collection, offering the potential for same-day diagnosis of enteric fever caused by *S*. Paratyphi A. Further development of the assay, such as multiplexing with primers specific for *S*. Typhi and/or nontyphoidal *Salmonella* may provide a valuable tool for detection of both typhoidal and nontyphoidal *Salmonella* infections.

## Material and Method

All procedures of the human paratyphoid challenge study were reviewed and approved by Oxford Research Ethics A Committee (Ref: 14/SC/0004) and conducted in accordance with the principles of the International Conference of Harmonisation Good Clinical Practice guidelines [24]. The blood samples used for assay optimisation in this study were obtained from healthy volunteers with written informed consent, in accordance with local ethically approved policies.

### Strains and Culture

The *S*. Paratyphi A strain NVGH308 was originally isolated in 2006 from a patient with acute paratyphoid fever by the Oxford University Clinical Research Unit at Patan Hospital, Kathmandu, Nepal. The strain was sub-cultured in tryptone soya broth (TSB) or on tryptone soya agar (TSA) (Oxoid, Basingstoke, UK) as needed. It has been manufactured to Good Manufacturing Practice (GMP) standard and was provided for use in a controlled human challenge study by Sclavo Behring Vaccines Institue for Global Health, Siena, Italy [24].

### Determination of growth of *S*. Paratyphi A Strain NVGH308 in TSB Ox Bile Medium

*S*. Paratyphi A strain NVGH308 was inoculated in 10 ml TSB, and incubated overnight in a 37°C incubator with shaking at 220 revolutions per minute (RPM). The overnight culture was then diluted 1:1,000 in 10 ml TSB, containing 0, 1.2, 2.4, 5.0, 10.0 or 15.0% (w/v) of ox bile (Difico^TM^ Oxgall, BD Biosciences, Oxford, UK), and further incubated at 37°C with shaking at 220 RPM for determination of growth of *S*. Paratyphi A. Bacterial growth was monitored during the course of 24 hours. The OD_600_ of each culture was recorded at 1.5 hour intervals from start to 7.5 hours and then at 24 hours to determine the growth characteristic of the strain.

### TSB Ox Bile Blood Culture of *S*. Paratyphi A Strain NVGH308

To determine an optimal duration of blood culture for PCR assay, a spiked culture was carried out with a modification of previously described methods [22]. Briefly, 2 ml of heparinised blood from healthy individuals was inoculated into 8 ml TSB containing 3.0% (w/v) ox bile in a 50 ml tube, producing a mixture of ox bile at 2.4% (w/v) and blood at 20% (v/v). The mixture was spiked with an average of 4 colony forming unit (CFU) of *S*. Paratyphi A strain NVGH308, and incubated at 37°C with shaking at 220 RPM for up to 5 hours. Two tubes were removed from the incubator every hour and the bacteria were collected by centrifugation at 5,000 **×** g for 20 min. The supernatant was discarded and the pellet was re-suspended in 0.2 ml phosphate-buffered saline. One tube was plated onto TSA plates for determination of bacterial CFU in each sample and another tube was used for DNA preparation.

### DNA Extraction

DNA was isolated from the culture using UltraClean™ BloodSpin™ Kit (MO BIO Laboratories, CA, USA) according to the manufacturer's instruction, except that the DNA was eluted with 50 μl buffer 5 preheated at 65°C and the filter unit was incubated at 65°C for 5 min before centrifugation. The aliquots (10 μl) of DNA preparation were used for PCR amplification.

### PCR Primers of *S*. Paratyphi A strain NVGH308

The PCR primers for *S*. Paratyphi A strain NVGH308 were designed according to the H-1a gene (*fliC*-*a*) for phase 1-a flagellin of *S*. Paratyphi A ATCC 9150 (Accession No: X03393): H-F (ACTCAGGCTTCCCGTAACGC), Ha-R1 (TGCCGTCTTTATCGGTATATTCAG) and Ha-R2 (GACTTCGCTCTTCACATCATAT), and synthesized by Sigma Genosys (Sigma-Aldrich, Dorset, England).

### PCR Protocol

The PCR reaction was carried out using TopTaq Master Mix Kit (Qiagen) in a 50 μl volume, comprising 25 μl of 2x TopTaq Master Mix, 5 μl of 10x CoralLoad Concentrate, 10 μl of 1 μM each of primers and 10 μl DNA template. The following amplification steps were used: 1cycle of 95°C for 5 min; 40 cycles of 93°C for 30 sec, 55°C for 30 sec, and 72°C for 40 sec; and 1 cycle of 72°C for 5 min. The PCR amplification product was separated by electrophoresis on a 1% agarose gel, stained with ethidium bromide, and analysed using the gel doc system Syngene G: Box.

### Paratyphoid Challenge and Blood Collection

Clinical specimens were obtained from healthy volunteers participating in a *S*. Paratyphi human challenge study. Study procedures are detailed elsewhere [24]. Briefly, forty healthy adult volunteers underwent oral challenge with *S*. Paratyphi A strain NVGH308 suspended in sodium bicarbonate, following pre-treatment with 2.1g sodium bicarbonate solution. Participants were challenged at dose levels 500–1,000 CFU or 1–5 x 10^3^ CFU. Pre-specified diagnostic criteria for paratyphoid fever were defined as follows: (1) A positive blood culture for *S*. Paratyphi A from 72 hours post-challenge; (2) A positive blood culture for *S*. Paratyphi A within 72 hours post-challenge, with one or more signs/symptoms of paratyphoid infection (such as recorded temperature ≥38°C); (3) Persistent positive blood cultures (two or more blood cultures taken at least 4 hours apart) for *S*. Paratyphi A within 72 hours post-challenge; and (4) Oral temperature ≥38°C persisting for 12 hours. On diagnosis of paratyphoid fever, 10 ml venous blood samples were taken for automated blood culture, 10 ml for quantitative blood culture, and 5 ml for blood culture PCR assay. Blood samples were collected for a variety of assays for the duration of the challenge period and limits to volume of blood that could be safely donated by study participants, prevented us from collecting equivalent volumes for all three assays.

### Blood Culture PCR Assay

For blood culture PCR assay of clinical blood samples, 5 ml venous blood was inoculated into 20 ml TSB containing 3.0% (w/v) ox bile and 1.5 μl (10^3^ gel units/μl) of micrococcal nuclease (New England Biolab, Herts, UK) in a 50 ml tube, producing a final concentration of ox bile at 2.4% (w/v) and blood at 20% (v/v). The culture was incubated at 37°C with shaking at 220 RPM for 5 hours, followed by centrifugation at 5,000 **×** g for 20 min to collect bacterial cells. The supernatant was discarded and the pellet was washed with 100 mM Tris, pH 8.0, 1 mM EDTA and then used for bacterial genomic DNA isolation. DNA was eluted in 50 μl volume. The presence of *S*. Paratyphi A was detected by PCR targeting the H-1a gene (*fliC-a*) for phase 1-a flagellin of *S*. Paratyphi A.

### Automated Blood Culture

Venous blood (10 ml) was cultured by direct inoculation into broth (BACTEC Plus Aerobic vials, BD) and subsequent automated growth detection (BD BACTECTM 9240 Blood Culture System), in accordance with standard methods [25]. *S*. Paratyphi growth and serotype were confirmed by biochemical profile (API-10S, bioMérieux, France) or slide agglutination according to the Kauffman-White classification, respectively [26].

### Quantitative Blood Culture

Quantitative blood culture was performed by using Wampole ISOSTAT/ISOLATOR Microbial System. Venous blood (10 ml) was lyzed by adding directly into an ISOLATOR 10 tube (Alere, UK). Following blood lysis, the tube was centrifuged for 30 min at 3,000 x g and the resulting pellet was plated onto XLD agar plate (Oxoid, UK) for isolation of bacteria. The plates were then incubated in a 37°C incubator for 24 hours prior to colony identification and counting.

## Results

### Effect of Ox Bile on the Growth of *S*. Paratyphi A Strain NVGH308 in TSB Medium

**Fig 1:**
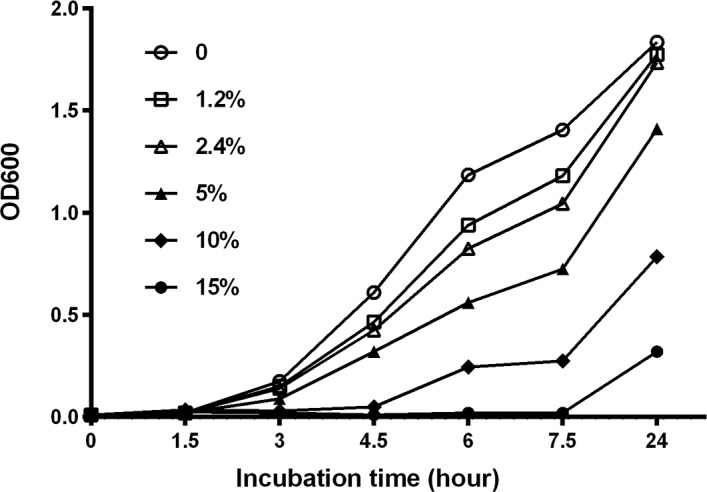
Effect of Ox Bile on the Growth of *S*. Paratyphi A Strain NVGH308 in TSB Ox Bile Medium. Overnight culture of *S*. Paratyphi A strain NVGH308 was inoculated 1:1,000 in TSB containing 0, 1.2, 2.4, 5.0, 10.0 or15.0% (w/v) of ox bile and grown in a 37°C incubator with shaking at 220 RPM. OD_600_ of each culture was recorded at 1.5 hour intervals. The OD_600_ value represents the mean ±SD of three independent cultures.

We have previously described that the addition of ox bile to TSB culture media causes rapid lysis of blood cells for release of intracellular bacteria without inhibiting the growth of *S*. Typhi, and improves assay sensitivity of a blood culture PCR method for identification of *S*. Typhi [22]. In this study we have investigated first the growth characteristics of S. Paratyphi A strain NVGH308 in TSB media at increasing concentrations of ox bile, ranging from 0 to 15%. The result is shown in Fig 1. Increasing concentrations of ox bile in the media inhibited the growth of *S*. Paratyphi A strain NVGH308. Using ox bile concentrations between 0 and 2.4%, terminal bacterial densities at 24 hours were similar despite some early retardation of growth; e.g. at 6 hours, the OD_600_ in 2.4% ox bile TSB medium was 0.825, compared with 1.185 without ox bile. At concentrations of ≥5.0%, ox bile supplementation markedly reduced the growth of *S*. Paratyphi A strain NVGH308, with an extended lag phase in bacterial growth seen at concentrations of 10% ox bile and above. No measurable bacterial growth occurred until the 4.5 hour timepoint and the 7.5 hour timepoint using 10% and 15% ox bile media respectively.

### Growth of *S*. Paratyphi A Strain NVGH308 in TSB—2.4% Ox Bile – 20% Blood Culture Medium

**Fig 2:**
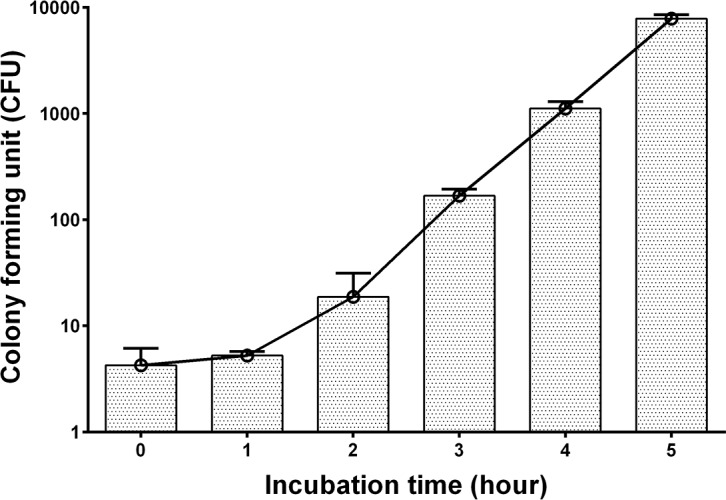
Growth of S. Paratyphi A Strain NVGH308 in TSB—2.4% Ox Bile – 20% Blood Culture Medium. An average of 4 CFU of bacteria was inoculated into 10 ml of TSB containing 2.4% ox bile and 20% blood, and incubated at 37°C with shaking at 220 RPM for up to 5 hours. Bacterial counts were determined every hour by plating onto TSA plates. The CFU count shown represents the mean ±SD of three independent inoculated blood cultures.

Based on the growth characteristics of *S*. Paratyphi A strain NVGH308 in TSB ox bile media, a TSB ox bile blood culture system containing 2.4% ox bile and 20% blood was selected for further development. The bacterial replication of *S*. Paratyphi A strain NVGH308 in this blood culture system was determined over a period of 5 hours and the result is shown in Fig 2.

Bacterial growth increased from the second hour of culture onwards. The number of CFU rose from 4 to 18 bacteria (2 generations) at 2 hours and 168 (about 5 generations) at 3 hours, and increased logarithmically at 5 hours of incubation. The bacteria underwent approximately 11 generations of replication during a 5 hour incubation period in the TSB—2.4% ox bile – 20% blood culture system, peaking at 7,800 CFU from a starting culture of an average of 4 CFU.

### PCR Optimization and Amplification

**Fig 3:**
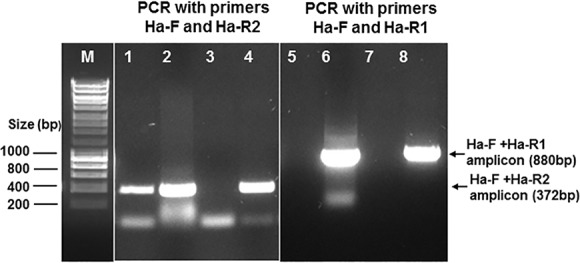
PCR primer optimization and amplification of the *fliC-a* gene of *S*. Paratyphi A. Lanes: M, DNA marker; 1 and 5, blood DNA only; 2 and 6, blood DNA spiked with *S*. Paratyphi A DNA; 3 and 7, no DNA negative control; 4 and 8, *S*. Paratyphi A DNA positive control.

Two pairs of PCR primers were designed and tested in PCR amplification using the blood DNA spiked with *S*. Paratyphi A DNA as template. We compared the primer pairs of H-F and Ha-R1 and H-F and Ha-R2 using the conditions described above. In the PCR conditions used, the primer pair of H-F and Ha-R2 produced an unspecific amplification of blood DNA in the absence of *S*. Paratyphi A DNA, with a size similar to that of the specific amplicon of *S*. Paratyphi A DNA (Fig 3, lane 1). In contrast, the primers H-F and Ha-R1 produced more specific amplification of the *fliC*-*a* gene for phase 1-a flagellin of *S*. Paratyphi A with a size of 880 bp, which was well distinguishable from those unspecific amplifications. The PCR amplicons were shown in Fig 3. Based on these findings the primer pair of H-F and Ha-R1 was selected for further development in the blood culture PCR assay in this study.

### Detection of *S*. Paratyphi A Using TSB—2.4% Ox Bile – 20% Blood Culture PCR Assay

**Fig 4:**
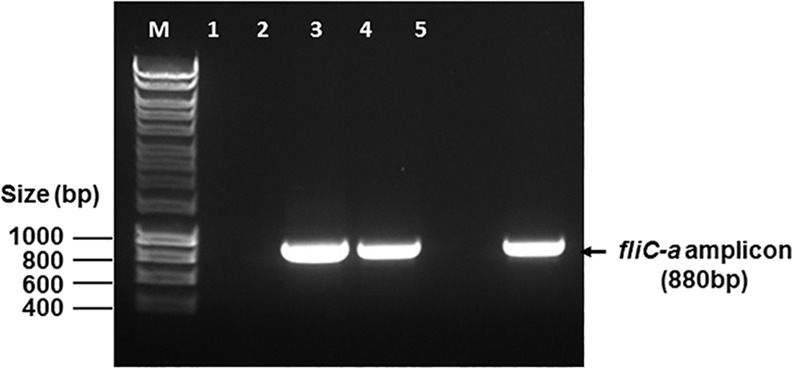
Detection of *S*. Paratyphi A using TSB—2.4% ox bile – 20% blood culture PCR assay. Lanes: M, DNA marker; 1, negative PCR control; 2 and 3, positive blood samples; 4, negative blood sample; 5, positive PCR control.

In order to determine the optimal culture duration of the blood culture PCR assay, DNA was isolated periodically from TSB—2.4% ox bile—20% blood culture spiked with 4 CFU of *S*. Paratyphi A and used for PCR amplification. The amplicons of *fliC-a* gene of *S*. Paratyphi A were consistently seen in PCR using the DNA preparations made from the cultures grown ≥ 3 hours, but were not seen in PCR using those prepared from the cultures grown for ≤ 2 hours (data not shown). When this blood culture PCR assay was applied to clinical paratyphoid samples, a culture with 5 ml blood sample was incubated for 5 hours prior to collection of bacteria for DNA preparation and subsequent PCR assay. A typical blood culture PCR result is shown in Fig 4.

### Performance of Blood Culture PCR Assay for Detection of *S*. Paratyphi A in A Human Challenge Model

Following challenge with *S*. Paratyphi A, 20/40 participants met pre-specified diagnostic criteria for paratyphoid fever (S1 Table), of whom 19/20 were diagnosed on the basis of a positive blood culture and 1/20 was diagnosed based upon persistent fever (temperature ≥38°C lasting ≥12 hours).

**Fig 5:**
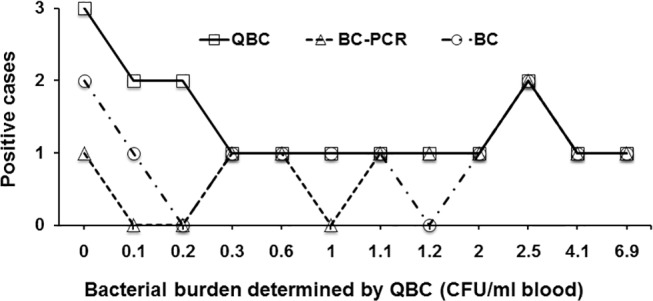
The bacterial burden together with the positivity of automated blood culture and blood culture PCR in 17 diagnosed paratyphoid participants. QBC: quantitative blood culture; BC-PCR: blood culture PCR; BC: automated blood culture.

Owing to variable sample collection, a complete set of blood samples was available in only 17/20 diagnosed participants for automated blood culture, quantitative blood culture and blood culture PCR assay. Blood culture PCR was positive in 10/17 (59%) samples collected immediately prior to commencement of antibiotics. In comparison, automated blood culture grew *S*. Paratyphi A in 12/17 (70%) cases and quantitative blood culture was positive in 14/17 (82%) individuals, with a median bacterial burden of 1 (range 0.1–6.9) CFU/ml blood. The bacterial burden together with the positivity of automated blood culture and blood culture PCR in the diagnosed paratyphoid participants is shown in Fig 5 and S2 Table. Direct comparison between each method is confounded by the fact that blood culture PCR was performed using half of the blood volume for automated and quantitative blood culture. Among the quantitative blood culture positive cases, 4 were negative by automated blood culture and 5 negative by blood culture PCR assay. Conversely, of 3/17 cases where quantitative blood culture did not show any bacterial growth, two were positive by automated blood culture and 1 was positive by blood culture PCR. Importantly, all the blood culture PCR positive cases where quantitative blood culture showed positive bacterial growth had a bacterial burden of ≥0.3 CFU/ml blood, suggesting that the lower limit of detection of the blood culture PCR assay was 0.3 CFU per ml blood.

We compared the performance of culture PCR assay with ‘gold-standard’ automated blood culture. Table 1 shows the performance of blood culture PCR assay and automated blood culture in the 17 paratyphoid participants diagnosed using the pre-specified criteria. Of 12 positive cases by automated blood culture, only 9 were also positive by blood culture PCR, while the latter tested 1 positive from the 4 cases where automated blood culture were negative. Both automated blood culture and blood culture PCR assay confirmed higher percentage of positive cases at the high bacterial burden (≥0.3 CFU/ml blood) of clinical specimens than at the low bacterial burden (≤0.2 CFU/ml blood). Additionally, the blood culture PCR assay identified the same number of positive cases (9/10) as the automated blood culture method at the bacterial burden of ≥0.3 CFU/ml blood, but fewer cases when a bacterial burden was ≤0.2 CFU/ml blood. Furthermore, blood culture PCR can be performed within 9 hours, instead of >36 hours required by automated blood culture, of time to detection (S3 Table) and identification of causative *S*. Paratyphi A.

**Table 1:** Performance of blood culture PCR assay and automated blood culture in participants diagnosed with paratyphoid fever (n = 17).

Category	Blood culture PCR assay		Automated blood culture	
	Positive	negative	Positive	Negative
**Automated blood culture positive (12cases)**	9/12	3/12		
**Automated blood culture negative (5 cases)**	1/5	4/5		
**Blood culture PCR assay positive (10 cases)**			9/10	1/10
**Blood culture PCR assay negative (7 cases)**			3/7	4/7
**Bacterial burden ≥0.3 CFU/ml blood (10 cases)**	9/10	1/10	9/10	1/10
**Bacterial burden ≤0.2 CFU/ml blood (7 cases)**	1/7	6/7	3/7	4/7
**Time taken from blood collection to identification of causative bacteria**	<9 hours		>36 hours*	

*Including median time to culture positivity 26.5 (18.5–44.7) hours and bacterial identification 18–24 hours.

## Discussion

In order to be introduced into routine clinical practice, any novel enteric fever diagnostic has to outperform the current ‘gold-standard’ automated blood culture, either in terms of cost, sensitivity, specificity, ease of use or rapidity of diagnosis. Despite over a decade of effort, PCR diagnostics of typhoid have not been brought into routine clinical use, partially due to the technical hurdles caused by a low bacterial load of clinical specimens [8]. In this study we describe a novel blood culture PCR method for detection of *S*. Paratyphi A, utilising an optimized TSB—2.4% ox bile culture system and a PCR assay targeting the *fliC-a* gene, and the steps taken to improve assay performance.

We went on to evaluate the performance of blood culture PCR assay using clinical specimens obtained from a unique *S*. Paratyphi challenge model in healthy volunteers. Blood culture PCR was positive in over 50% of participants who met the pre-specified diagnostic criteria for paratyphoid fever, and performed well compared with the current gold-standard diagnostic method.

DNA template preparations from specimens containing a low bacterial load (such as that seen in typhoid and paratyphoid infection) are dominated by the background host blood DNA, which swamps the bacterial target signal leading to either false-positive PCR results (due to the non-specific binding of primer) or false-negative results due to reduced sensitivity [19–21]. In order to overcome this limitation, we and others previously utilized a blood culture to enrich *S*. Typhi bacteria from clinical specimens prior to PCR detection, which improves the sensitivity of the PCR assay and increases the rate of detection [22,23]. Paratyphoid, like typhoid, has a low bacterial burden during infection. We have demonstrated in this study that the bacterial load varied from 0.1 to 6.9 (a median of 1) CFU per ml blood among the participants diagnosed with paratyphoid on the day of diagnosis. This finding affirms that a bacterial pre-enrichment step is necessary in order to improve the sensitivity of PCR assay for detection of *S*. Paratyphi in clinical specimens of paratyphoid. In addition, we found that the addition of micrococcal nuclease to TSB – 2.4% ox bile culture broth dramatically reduces background host DNA, overcoming the technical problem in bacterial DNA isolation, increasing the relative quantity of bacterial DNA and potentially enhancing PCR sensitivity and specificity.

Both *S*. Typhi and *S*. Paratyphi are intracellular pathogens. More than 50% of the bacteria are concentrated within cells in blood and bone marrow of typhoid patients [9]. Additionally, evidence indicates that human blood has bactericidal activity against *Salmonella* [26–28]. A culture medium which is able to lyze blood cells for the release of intracellular bacteria and inhibit the bactericidal activity of blood would be beneficial to developing a fast blood culture PCR assay system. Historically, bile has been used as a component of culture media for isolation of enteric pathogens such as *S*. Typhi and *S*. Paratyphi from whole blood, utilising its capacity to cause cellular lysis and to inhibit bactericidal activity of blood [29]. We previously demonstrated that TSB media containing ox bile is suitable for *S*. Typhi enrichment [22]. We have investigated it further in this study for enrichment of *S*. Paratyphi A, and describe herein the growth characteristics of *S*. Paratyphi A strain NVGH308 at increasing concentrations of ox bile, ranging from 0 to 15%. Terminal bacterial densities at 24 hours were similar when using ox bile from 0 to 2.4%, despite some early retardation of growth with the increase of bile concentration. A concentration of 5.0% or higher of ox bile markedly reduced the growth of *S*. Paratyphi A strain NVGH308. The growth behaviour of *S*. Paratyphi A in ox bile containing TSB media was similar to that of S. Typhi reported previously [22]. We previously reported a fast and sensitive blood culture PCR assay for S. Typhi utilizing a TSB-2.4% ox bile broth with 20% blood in which *S*. Typhi was able to replicate by 10 generations within 5 hours of incubation [22]. We conclude that the same broth as used for *S*. Typhi is optimal for pre-enrichment of *S*. Paratyphi A in a blood culture PCR assay. The *S*. Paratyphi A strain NVGH308 growth, indeed like *S*. Typhi, in this culture system was approximately 5 to 11 generations within 3–5 hours of incubation.

A potential advantage of the culture PCR technique described here is the ability to offer rapid diagnosis of enteric fever–with appropriately trained staff, the entire assay (blood culture, DNA preparation and PCR detection) can be performed within 9 hours, allowing for the same day diagnosis and treatment initiation for paratyphoid. A key to reduction of assay time in blood culture PCR is the incubation period of blood culture. As was shown in our previous study, a 5 hour TSB-2.4% ox bile blood culture PCR assay could detect *S*. Typhi as low as 0.75 CFU/ml in blood [22], and increased the speed of a positive confirmatory diagnosis of typhoid in a human challenge study [8]. Recently, another study used a 6 hour BHI broth culture for bacterial enrichment in PCR detection of blood typhoidal and non typhoidal *Salmonellae* infections [23]. Based on the similarity in growth between *S*. Paratyphi A and *S*. Typhi in ox bile containing TSB media as discussed above, we conclude that 5 hour incubation of a blood culture in TSB-2.4% ox bile broth represents a compromise between speed and sensitivity of the blood culture PCR assay when applied to clinical specimens (5 ml) of enteric paratyphoid fever.

In 2011, we established an *S*. Typhi human challenge model with the aim of creating a platform to understand disease pathogenesis, and assess novel vaccine candidates and novel diagnostics [30]. In a similar fashion, we have subsequently developed the first ever *S*. Paratyphi A human challenge model, which offers the unique opportunity to assess the performance of the blood culture PCR assay in clinically relevant samples in a highly controlled setting [24]. Using samples collected from 17 participants diagnosed with paratyphoid fever immediately before commencement of antibiotics, we attempted to compare the performance of the blood culture PCR assay with an automated blood culture (gold standard) method. It should be noted that the blood culture PCR assay used only half the volume of specimens for the automated blood culture in this study, which was due to the compromise to reduce the total blood volume taken from volunteers as a number of assays were being performed at paratyphoid diagnosis. The blood culture PCR detected overall fewer positive cases than the automated blood culture in this study. However, when comparing the positivity of assays among the samples having a bacterial burden of ≥0.3 CFU/ml blood, we found that the blood culture PCR assay performed as well as the automated blood culture, both detecting 9/10 positive cases, although the blood culture PCR used only half the blood volume for the automated blood culture. Additionally we found that all the blood culture PCR positive cases where the quantitative blood culture showed a positive bacterial growth had a bacterial burden of ≥0.3 CFU/ml blood, indicating that the lower limit of detection of blood culture PCR assay was 0.3 CFU per ml blood. This finding has for the first time demonstrated quantitatively the sensitivity of a PCR assay which is related to a bacterial load in the clinical specimens of enteric fever. As the volume of blood used in this study was limited by constraints on sample volume of collection, we suggest that the sensitivity of the blood culture PCR assay could be improved by using a large volume blood specimen e.g. 10 ml. As mentioned above, *S*. Typhi and *S*. Paratyphi can replicate by up to 10–11 generations in the TSB-2.4% ox bile blood culture broth in 5 hours. Using a 10 ml blood specimen having a bacterial burden of 0.1 CFU/ml, a total bacterial CFU will amount to ≥1,024 following 5 hours of incubation. We previously showed 5 CFU per PCR reaction was the minimum detection limit of conventional PCR [22]. Therefore, the blood culture PCR assay described herein, if it had used the same volume (10 ml) of blood as the automated blood culture in this study, would have probably confirmed all the positive cases of the automated blood culture as long as a 10 ml blood sample contained 1 bacterium (0.1 CFU/ml). However, it is likely that sampling effect caused by an intrinsically low bacterial burden of enteric fever, such as occurred in this study that some positive samples by quantitative blood culture were negative by automated blood culture, and *vice versa*, could produce a false-negative PCR result.

Infection with *S*. Typhi and *S*. Paratyphi causes an almost indistinguishable clinical syndrome. The trend towards increasing incidence of paratyphoid infection in several endemic countries, particularly in South Asia, makes the development of rapid diagnostics that are able to detect both *S*. Typhi and *S*. Paratyphi desirable [31] The blood culture PCR assay described could be multiplexed to detect both S. Typhi and *S*. Paratyphi, and potentially invasive nontyphoidal *Salmonella* infections, which have emerged as a prominent cause of bloodstream infection in African adults and children, with an associated case fatality of 20–25% [32].

A variety of PCR-based techniques are currently in development for the diagnosis of typhoid and paratyphoid fever, including multiplex PCR [16], real-time PCR [18], LAMP based technique [33]. Whilst many of these techniques offer distinct advantages, all are limited by the low bacterial burden found in blood during acute enteric fever. We believe that the blood culture PCR technique can overcome some of these limitations and could be adapted in the development of similar assays. Potential limitations of blood culture PCR method include low sensitivity, which could be mitigated by performing the assay with a larger sample volume. Like automated blood culture, antibiotic use prior to blood collection abates the detection rate of blood culture PCR method. Additionally, the blood culture PCR technique is not fully automated and despite being potentially faster than automated blood culture, it remains relatively labour intensive and requires specialist equipment and trained staff who are familiar with the techniques used. It does not negate the need for additional staff and dedicated facilities, and further economic analysis is required to assess if the technique is cost-effective compared to the current gold-standard. Nevertheless, we believe these findings in this study warrant further study of the blood culture PCR assay, particularly in endemic populations.

## Conclusions

The blood culture PCR described in this study takes under 9 hours and offers the potential for same-day diagnosis of enteric fever. Using 5 ml blood, it exhibited a lower limit of detection of 0.3 CFU/ml blood, comparable to the actual bacterial load of clinical samples from endemic populations, and it performed at least as well as automated blood culture at higher bacterial loads (≥0.3 CFU/ml blood) of clinical specimens despite using half the volume of blood. The findings warrant its further study in endemic populations with a potential use as a novel diagnostic which fills the present gap of paratyphoid diagnostics. In addition, this novel blood culture PCR assay could be potentially multiplexed as a valuable tool for detection of both typhoidal and nontyphoidal *Salmonella* infections.

## Supporting Information

S1 Table Participants challenged with S. Paratyphi and clinical diagnosis of paratyphoid. (DOC)

S2 Table Automated blood culture, quantitative blood culture and blood culture PCR assay on the diagnosis day of paratyphoid. (DOC)

S3 Table Time to positivity of automated blood culture of the samples collected on the diagnosis day of paratyphoid. (DOC)
