# Supplementary material for: Development and Evaluation of a Blood Culture PCR Assay for Rapid Detection of Salmonella Paratyphi A in Clinical Samples
Source: PLoS One. 2016 Mar 1;11(3):e0150576. doi: 10.1371/journal.pone.0150576 (PMC4773247; doi:10.1371/journal.pone.0150576)
Supplement: S1 Table — (DOC) [file pone.0150576.s001.doc]

**S1 Table. Participants challenged with *S*. Paratyphi and clinical diagnosis of paratyphoid**

| **Challenge dose** | **Participant ID** | **Clinical paratyphoid** | **Clinical diagnosis criteria** |
| --- | --- | --- | --- |
| 1-5 x 10^3^ CFU | 0054 | 1 | BC |
|  | 0072 | 1 | BC |
|  | 0070 | 0 |  |
|  | 0077 | 0 |  |
|  | 0014 | 1 | BC |
|  | 0019 | 0 |  |
|  | 0071 | 1 | BC |
|  | 0002 | 1 | BC |
|  | 0042 | 0 |  |
|  | 0053 | 1 | BC |
|  | 0035 | 0 |  |
|  | 0076 | 0 |  |
|  | 0027 | 1 | BC |
|  | 0043 | 1 | BC |
|  | 0056 | 0 |  |
|  | 0011 | 1 | Temperature |
|  | 0037 | 0 |  |
|  | 0049 | 1 | BC |
|  | 0036 | 1 | BC |
|  | 0059 | 1 | BC |
| 500-1,000 CFU | 0006 | 1 | BC |
|  | 0016 | 0 |  |
|  | 0024 | 0 |  |
|  | 0013 | 0 |  |
|  | 0080 | 1 | BC |
|  | 0050 | 0 |  |
|  | 0012 | 0 |  |
|  | 0047 | 0 |  |
|  | 0058 | 1 | BC |
|  | 0028 | 0 |  |
|  | 0067 | 0 |  |
|  | 0029 | 1 | BC |
|  | 0001 | 1 | BC |
|  | 0073 | 0 |  |
|  | 0064 | 0 |  |
|  | 0079 | 1 | BC |
|  | 0025 | 0 |  |
|  | 0008 | 1 | BC |
|  | 0034 | 0 |  |
|  | 0022 | 1 | BC |
| BC: Automated blood culture; 1 and 0: Positive and negative case based on predefined criteria, respectively | | | |
