# Supplementary material for: Development and Evaluation of a Blood Culture PCR Assay for Rapid Detection of Salmonella Paratyphi A in Clinical Samples
Source: PLoS One. 2016 Mar 1;11(3):e0150576. doi: 10.1371/journal.pone.0150576 (PMC4773247; doi:10.1371/journal.pone.0150576)
Supplement: S2 Table — (DOC) [file pone.0150576.s002.doc]

**S2 Table.** Automated blood culture, quantitative blood culture and blood culture PCR assay on the diagnosis day of paratyphoid

| **Blood volume** | **10 ml** | **10 ml** | **5 ml** |
| --- | --- | --- | --- |
| **Participant ID** | **BC** | **BQ CFU/ml** | **BC-PCR PCR** |
| 0054 | 1 | 1.1 | 1 |
| 0072 | 1 | 2.6 | NA |
| 0014 | 1 | 0.6 | 1 |
| 0071 | 1 | 0 | 0 |
| 0002 | 1 | 0.3 | 1 |
| 0053 | 1 | 2.5 | 1 |
| 0027 | NA | NA | NA |
| 0043 | 0 | 1.2 | 1 |
| 0011 | 1 | 0.1 | 0 |
| 0049 | 1 | 1 | 0 |
| 0036 | 1 | 4.1 | 1 |
| 0059 | 1 | 1.8 | NA |
| 0006 | 1 | 2.5 | 1 |
| 0080 | 0 | 0.1 | 0 |
| 0058 | 0 | 0.2 | 0 |
| 0029 | 1 | 0 | 1 |
| 0001 | 1 | 2 | 1 |
| 0079 | 1 | 6.9 | 1 |
| 0008 | 0 | 0.2 | 0 |
| 0022 | 0 | 0 | 0 |
| BC: Automated blood culture; BQ: quantitative blood culture; BC-PCR: Blood culture PCR assay; 1: positive; 0: negative; NA: sample is not available | | | |
