# Supplementary material for: Development and Evaluation of a Blood Culture PCR Assay for Rapid Detection of Salmonella Paratyphi A in Clinical Samples
Source: PLoS One. 2016 Mar 1;11(3):e0150576. doi: 10.1371/journal.pone.0150576 (PMC4773247; doi:10.1371/journal.pone.0150576)
Supplement: S3 Table — (DOC) [file pone.0150576.s003.doc]

**S3 Table. Time to positivity of automated blood culture of the samples collected on the diagnosis day of paratyphoid**

| **Participant ID** | **Date of diagnosis** | **Date/Time Collected** | **Date/Time Positive** | **Time to Positivity (Hr:Min:Sec)** |
| --- | --- | --- | --- | --- |
| 054 | 26 May 2014 | 26/05/2014 18:30 | 27/05/2014 20:55 | 26:25:00 |
| 072 | 13 June 2014 | 13/06/2014 18:00 | 14/06/2014 17:24 | 23:24:00 |
| 071 | 02 July 2014 | 02/07/2014 10:00 | 03/07/2014 12:38 | 26:38:00 |
| 014 | 11 June 2014 | 11/06/2014 18:10 | 13/06/2014 02:01 | 31:51:00 |
| 002 | 02 July 2014 | 02/07/2014 14:47 | 04/07/2014 00:13 | 33:26:00 |
| 053 | 02 July 2014 | 02/07/2014 21:33 | 04/07/2014 00:03 | 26:30:00 |
| 027 | N/A | N/A | N/A |  |
| 043 | 06 August 2014 | 06/08/2014 09:45 | 07/08/2014 08:20 | 22:35:00 |
| 011 | 07 August 2014 | 07/08/2014 07:00 | 08/08/2014 14:06 | 31:06:00 |
| 036 | 06 August 2014 | 06/08/2014 08:08 | 07/08/2014 06:31 | 22:23:00 |
| 049 | 06 August 2014 | 06/08/2014 14:10 | 07/08/2014 12:55 | 22:45:00 |
| 059 | 08 August 2014 | 08/08/2014 08:40 | 10/08/2014 05:23 | 44:43:00 |
| 006 | 23 October 2014 | 23/10/2014 10:00 | 24/10/2014 18:46 | 32:46:00 |
| 080 | 23 October 2014 | 23/10/2014 10:20 | N/A | N/A |
| 058 | 02 November 2014 | 02/11/2014 09:15 | N/A | N/A |
| 029 | 21 November 2014 | 21/11/2014 06:15 | 22/11/2014 12:09 | 29:54:00 |
| 001 | 20 November 2014 | 20/11/2014 15:45 | 21/11/2014 10:13 | 18:28:00 |
| 079 | 27 November 2014 | 27/11/2014 10:10 | 28/11/2014 05:19 | 19:09:00 |
| 008 | 28 November 2014 | 28/11/2014 17:00 | N/A | N/A |
| 022 | 02 December 2014 | 02/12/2014 09:45 | N/A | N/A |
